# System Dynamic Model Simulates the Growth Trend of Diabetes Mellitus in Chinese Population: Implications for Future Urban Public Health Governance

**DOI:** 10.3389/ijph.2022.1605064

**Published:** 2022-11-11

**Authors:** Hao Li, Guo-Ying Chang, Yi-Hong Jiang, Li Xu, Long Shen, Zhi-Chun Gu, Hou-Wen Lin, Fang-Hong Shi

**Affiliations:** ^1^ Clinical Research Ward, Clinical Research Center, Shanghai Children’s Medical Center, School of Medicine, Shanghai Jiao Tong University, Shanghai, China; ^2^ China Institute for Urban Governance, Shanghai Jiao Tong University, Shanghai, China; ^3^ Department of Endocrinology, Shanghai Children’s Medical Center, School of Medicine, Shanghai Jiao Tong University, Shanghai, China; ^4^ Department of Endocrinology, Ren Ji Hospital, Shanghai Jiao Tong University School of Medicine, Shanghai, China; ^5^ Department of Cardiology, Ren Ji Hospital, Shanghai Jiao Tong University School of Medicine, Shanghai, China; ^6^ Department of Pharmacy, Ren Ji Hospital, Shanghai Jiao Tong University School of Medicine, Shanghai, China

**Keywords:** diabetes, type 2 diabetes, prevention strategies, economic burden, Chinese population, health policies, system dynamic model, urban public health governance

## Abstract

**Objectives:** To simulate the growth trend of diabetes mellitus in Chinese population.

**Methods:** The system dynamic modeling methodology was used to establish a population prediction model of diabetes with or without cardiovascular diseases. Lifestyle therapy and the use of metformin, acarbose, and voglibose were assumed to be intervention strategy. The outcomes will be examined at 5, 15, and 30 years after 2020.

**Results:** The projected number of diabetic population in China would increase rapidly from 141.65 million in 2020 to 202.84 million in 2050. Diabetic patients with cardiovascular disease would rapidly increase from 65.58 million in 2020 to 122.88 million by 2050. The annual cost for the entire population with diabetes mellitus in China would reach 182.55 billion by 2050. When the treatment of cardiovascular disease was considered, expenditure was 1.5–2.5-fold higher. Lifestyle therapy and the use of metformin, acarbose and voglibose could effectively slow the growth of the diabetic population.

**Conclusion:** The diabetic population in China is expected to increase rapidly, and diabetic patients with cardiovascular disease will increase greatly. Interventions could delay it.

## Introduction

Diabetes mellitus (DM) is a complex chronic disease that requires continuous medical care [1]. China has the largest number of patients with DM globally, accounting for one in four all adults living with DM worldwide [2]. Over the recent decades, the prevalence of DM in China has increased from 1% in the 1980s to 5.5% in 2001, 9.7% in 2007, 11.6% in 2010, and 12.8% in 2017 [3, 4]. The extensive national survey indicated that the tota population with DM in mainland China is estimated to be 129.8 million in 2017 [4], and is expected to increase to 174.4 million by 2045 [5]. Owing to the rapid growth of the population with DM, the increasing cost of its prevention and control has increased the health burden [4]. It is estimated that the DM-related health expenditure in China will be US$ 165.3 billion in 2021 [5]. Accordingly, China will face a serious burden of diabetes prevention and control in the future.

Interventions can be advanced according to the prediction results only by accurately predicting the future situation. Scholars in the United States [6] and the Netherlands [7] have made several future projections of diabetes burden and total prevalence in their own countries. The International Diabetes Federation (IDF) Diabetes Atlas 10th edition provided country-level estimates on DM prevalence and health expenditures, which projected that the population with DM in China would reach over 174 million by 2045 [5]. However, some large epidemiological data [8, 9] and data from intervention studies [10, 11] in China were not included into their prediction model. To date, no prediction has focused on DM population and expenditure in China.

The prevention and treatment of diabetes and its complications is dynamic and complex [12]. Early and appropriate intervention can significantly affect the occurrence, development, and deterioration of DM and its complications [13]. Lifestyle therapy and drug therapy such as metformin [14], acarbose [15], and voglibose [16], can delay the progression of DM. However, the overall impact of these interventions on the future population with DM in China remains unknown. Simulation models are considered sensible choices for solving complex problems [17].

The system dynamics (SD) model, which combines social science and behavioral science, can simulate complex dynamic behavior over time *via* interlocking sets of differential and algebraic equations developed from an extensive spectrum of relevant empirical data [18]. The main features of SD simulation are a feedback loop, stocks and flow function, and time delay, and it can be carried out qualitatively or quantitatively [17]. It has been effectively used to address dynamically complex issues in healthcare [17, 19], health policy [20] and the management of chronic diseases [21, 22], including DM [7]. Currently, scholars from the United States and the Netherlands have reported the simulating results of population with DM by SD models [7, 23]. However, these models cannot be directly applied to the Chinese population. In the present study, we optimized an SD model of the journey of patients with diabetes to visualize and explore the consequences *via* different intervention scenarios. Meanwhile, we also introduced an important complication of DM, cardiovascular disease (CVD), into our SD model [24]. We hope to use SD models to simulate the control of different interventions on the DM population and health expenditures to provide a theoretical tool for future decision-makers to formulate intervention strategies.

## Methods

### The Model Structure

**FIGURE 1:**
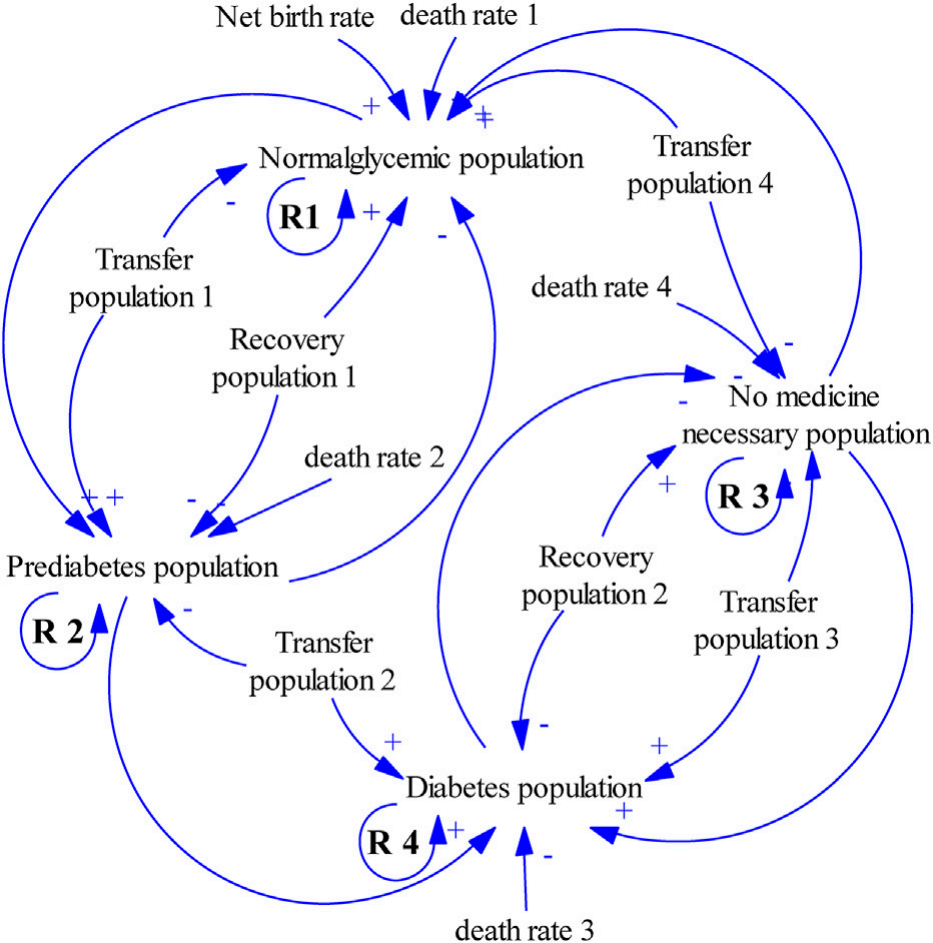
Simple casual loop diagram. (System dynamic model simulates the growth trend of diabetes mellitus, China. 2020–2050). The four stocks are the normoglycemic, prediabetes, no necessary medicine, and diabetes populations. People with normal blood glucose and those who do not need medication will develop prediabetes, and then diabetes. Few people with diabetes will return to a state that does not require drugs, and some people with prediabetes will return to standard blood glucose levels. The higher the baseline population in the four states, the higher the number of people, and a reinforcing loop (R1 to R4) is presented. Arrows and plus signs indicate positive effects, whereas arrows with negative signs show negative effects.

The current SD model was derived from the original diabetes model of the Center for Diabetes Control and Prevention in the USA [25] and recent research on the Dutch population [7]. The modeling methodology of SD was used to create simulation models for evaluating the impact of different prevention strategies on the DM population, diabetic patients with or without CVD, and the total health cost in China. A simplified causal loop diagram and its behavior, illustrating stocks and flows, are shown in Figure 1. A population model and its behavior illustrate stocks and flows, in which numbers of people in different states are called “stocks” (e.g., DM population, prediabetes population), and the numbers transitioning between the states are called “flows” (e.g., undiagnosed prediabetes to DM, diagnosed prediabetes to DM). The variables are indicated by words preceding single arrows, adding detailed information between stocks and flows. Arrows and plus signs indicate positive effects, whereas arrows with negative signs show negative effects.

The model consists of two linked sub-models: the DM model (Model 1) and the combined CVD model (Model 2). Both models are used to estimate the progress of DM population and the effect of different interventions on the progress of DM population. The models presented herein were developed and modified in four stages: 1) participatory systems mapping and conceptual diagram development based on prior diabetes models and expert consensus [7, 25]; 2) converting the conceptual diagram into a computational model *via* parameterization with numeric inputs (mainly data from Chinese studies); 3) design, integrate, test, and optimize the strategies; and 4) model validation and intervention studies. The specific method for model establishment can be found in previous studies [7, 25]. Compared with previous studies, we do not distinguish between types of diabetes, but we differentiated the DM population combined with CVD [7, 25].

**FIGURE 2:**
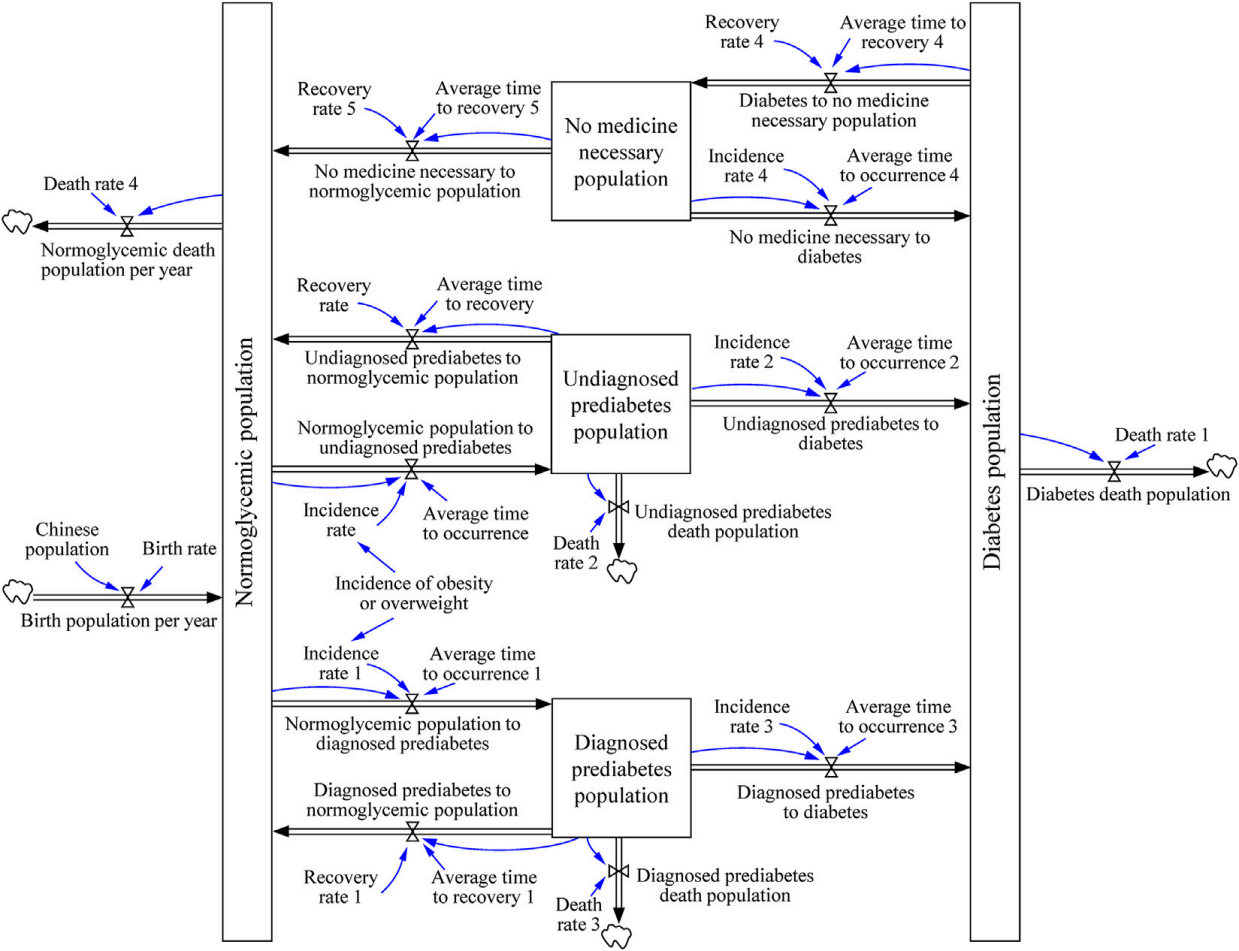
The journey of patient with diabetes. (System dynamic model simulates the growth trend of diabetes mellitus, China. 2020–2050). Boxes are stocks that accumulate over time, and double arrows are in- or outflows that either provide input or take the output of stocks. The blue arrows indicate the links between cause and effect. Clouds are outputs that “flow out of the system” like death. The system contains the differential equations described in the “Building the DM Model” section.

The DM model was used to project the growth trend of DM population in China. The model structure settings are shown in Figure 2. We assumed that the population related to DM was divided into three categories: normoglycemic, prediabetes, and DM populations. The state of the normoglycemic population was defined as fasting blood glucose <6.1 mmol/L and 2-hour postprandial blood glucose <7.8 mmol/L. Prediabetes population were individuals with fasting blood glucose between 6.1 and 7.0 mmol/L (impaired fasting glucose, IFG), or 2-hour postprandial blood glucose between 7.8 and 11.1 mmol/L (impaired glucose tolerance, IGT), or both conditions are satisfied. Likewise, the DM population referred to individuals with fasting blood glucose >7.0 mmol/L or 2-hour postprandial or random blood glucose >11.1 mmol/L [26]. DM population leaves the DM stocks in two ways: either they die due to DM when reaching the average age of DM patients, or by recovering from DM.

**FIGURE 3:**
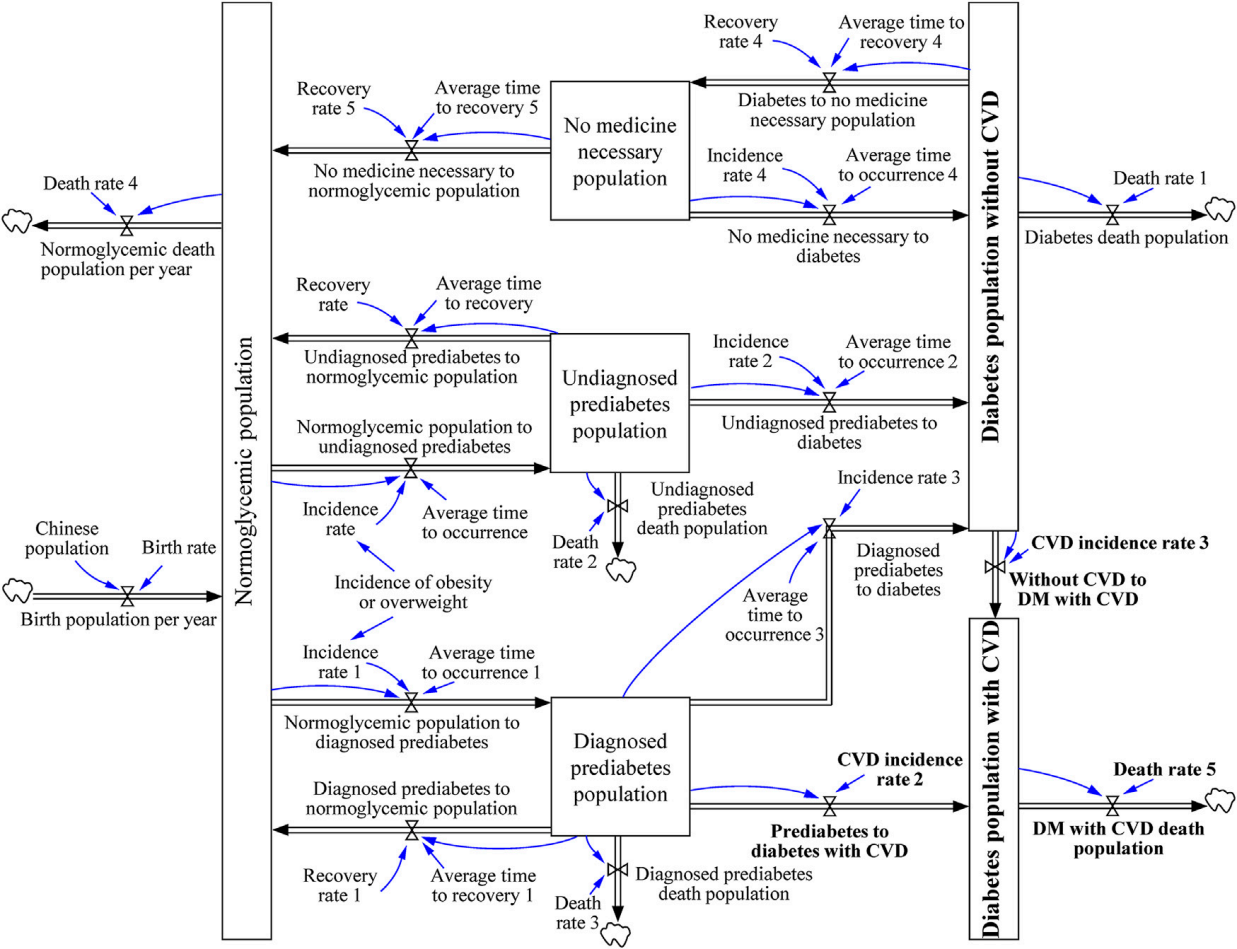
The journey of diabetic patient with cardiovascular disease. (System dynamic model simulates the growth trend of diabetes mellitus, China. 2020–2050). The journey was modified from “the journey of patient with diabetes,” which divided the population with diabetes into diabetic population without cardiovascular disease and diabetic population with cardiovascular disease. The system contains the differential equations described in the “Building the DM combined CVD Model” section.

In Model 2, we introduced CVD, the most noticeable complication of DM. The difference between the two models was that in Model 2 the diabetic population was divided into two categories: DM with or without CVD (Figure 3). DM is associated with a higher risk of CVD [27, 28]. In this study, we hypothesis that diabetes occurs first and then CVD. Indeed, people with CVD could also later develop type 2 diabetes, especially considering many similar risk factors are shared by these two diseases [27]. As we only obtain the epidemiological data of CVD in diabetes patients. Therefore in this study, we only included the population who developed CVD after DM but not those who developed CVD before DM.

### Interventions and Scenarios

Lifestyle therapy and the use of metformin and acarbose are the main strategies recommended for the prevention of prediabetes by the American Association of Clinical Endocrinologists and American College of Endocrinology [29]. Considering the cardiovascular risk benefits of α-glucosidase inhibitors, we introduced another α-glucosidase inhibitor, voglibose, into our models to predict its effect on DM control. The four strategies and detailed parameter values used are listed in Table 1. Finally, five scenarios, including no intervention, lifestyle therapy and the use of metformin, acarbose, and voglibose were substituted into the models for simulation.

**TABLE 1:** Different intervention scenarios and their parameter values used in scenario simulation (Parameter values were obtained from Chinese census data, Epidemiological data, Da Qing Diabetes Prevention study, and several studies on health economics, China, and other countries. 1980–2019).

	No intervention	Lifestyle	Metformin	Acarbose	Voglibose
DM model (model 1)					
Diagnosed prediabetes to diabetes	0.113 (1y) [10]	0.57 (0.41–0.81) [10]	0.69 (0.57–0.83) [37]	0.75 (0.63–0.90) [38]	0.60 (0.43–0.82) [16]
Undiagnosed prediabetes to diabetes	0.083 (1y) [7,10]	0.57 (0.41–0.81) [10]	0.69 (0.57–0.83) [37]	0.75 (0.63–0.90) [38]	0.60 (0.43–0.82) [16]
Undiagnosed prediabetes to normoglycemic population	0.322 (6y) [32]	—	—	1.14 (0.97–1.32) [38]	1.54 (1.36–1.75) [16]
Diagnosed prediabetes to normoglycemic population	0.322 (6y) [32]	—	—	1.14 (0.97–1.32) [38]	1.54 (1.36–1.75) [16]
Death rate	0.0170 [10]	0.0143 [11]	0.93 (0.88–0.99) [37]	—	—
DM combined CVD model (model 2)					
Prediabetes to diabetes with CVD rate	0.0259 [32]	0.48 (0.40–0.59) [34]	0.76 (0.66–0.87) [14]	0.66 (0.64–0.68) [15]	1.24 (0.82–1.86) [36]
Diabetes without CVD to CVD rate	0.0391 [32]	0.48 (0.40–0.59) [34]	0.76 (0.66–0.87) [14]	0.66 (0.64–0.68) [15]	1.24 (0.82–1.86) [36]
Cost (US$, per patient per year)					
Cost for screening	3 [33]	3 [33]	3 [33]	3 [33]	3 [33]
Onset of diabetes	897 [35]	897 [35]	897 [35]	897 [35]	897 [35]
CVD treatment	2078 [35]	2078 [35]	2078 [35]	2078 [35]	2078 [35]
Lifestyle therapy (Diet and exercise)	—	371 [33]	—	—	—
Metformin therapy	—	—	163 [37]	—	—
Acarbose therapy	—	—	—	185 [38]	—
Voglibose therapy	—	-	—	—	242 [16]

In models 1 and 2, data are presented as incidence rate per year or RR with a confidence interval. RR, risk ratio; CVD, cardiovascular disease. The prices of metformin, acarbose, and voglibose were collected from the YAOZHI database (https://db.yaozh.com) based on clinical trials.

### Parameter Setting

The demographic dataset used as the baseline Chinese population was obtained from the Chinese census data (National Bureau of Statistics). Epidemiological data on diabetes in China were obtained mainly from four large epidemiological surveys conducted in 2007 [8], 2010 [30], 2013 [31], and 2017 [4]. The conversion rates from prediabetes to DM and from DM to CVD were collected from the Da Qing Diabetes Prevention study [10, 11, 32]. The cost of DM expenditure, which includes DM prevention and treatment, was obtained from a societal perspective [33]. The cost for DM patients with CVD included the expenditure on CVD prevention and treatment [34, 35]. The cost of lifestyle interventions was based on a published modeling study [33]. The cost of drug intervention strategies was mainly calculated from the price of each drug (YaoZhi database, https://db.yaozh.com/) and the drug dosage in the intervention programs (metformin, acarbose, or voglibose) [16, 33, 36–38]. A schematic overview of the total cost of diabetes using dynamic simulations is shown in Supplementary Figure S1. The input values for the models used in this study are presented in Supplementary Tables S1–S3.

### Outcomes

DM population, DM population with or without CVD, and total cost of DM were used as the outcomes of this study. The time horizon of the model was 30 years from intervention implementation, based on proposals of party leadership for formulating the 14th Five-Year Plan for National Economic and Social Development and the Long-Range Objectives, through the year 2035. The outcomes were examined at 5, 15, and 30 years.

### Validation of the Model

The representations of the model equations underwent repeated checks with the modelling alliance for structural verification. The data and equations for these models are presented in Supplementary Tables S1, S2. To validate the model, the initial value of the variables was set to 2007, which was simulated in the next 10 years (2007–2017). Key predicted DM population outcomes were used for calibration against administrative data recorded in published diabetes epidemiology studies in 2007 [8], 2010 [30], 2013 [31] and 2017 [4]. To parameterize the model, data were verified against published evidence and data, or consensus within the modeling alliance in the absence of directly relevant data. A stock-flow diagram was established using the Vensim PLE software package (7.3.5) based on the system boundaries, inputs, outputs, and auxiliary variables.

## Results

### Model Validation

The validation results are presented in Table 2. The behavior reproduction test examined the ability of model to replicate the reference behavior model on published data on the DM population from 2007 to 2017. The mean absolute percentage error (MAPE) ranged from 1.22 to 5.47 (average, 2.33) and 0.77–5.77 (average, 2.17) in the DM and DM combined CVD models, respectively, which is a reasonable range. Thus, the model is considered valid and reasonable.

**TABLE 2:** Validation analysis of the model on population with diabetes (Million) (System dynamic model simulates the growth trend of diabetes mellitus, China. 2007–2017).

Time (Year)	Actual data	Model 1	Model 2	MAPE 1	MAPE 2
2007	92.4	92.4	92.4	0.00	0.00
2010	113.9	107.7	107.3	5.47	5.77
2013	118.5	119.9	119.4	1.22	0.77
2017	129.8	133.2	132.6	2.63	2.13
Average MAPE	—	—	—	2.33	2.17

Actual data are mainly based on published epidemiological data in China; Model 1 represents the diabetes journey and Model 2 represents diabetes combined with CVD journey. MAPE1 and MAPE2 indicate the results of Model 1 and Model 2, respectively. MAPE, mean absolute percentage error.

### Projections of DM Population

The projections of the number of patients with DM and DM with or without CVD from 2020 to 2050 are presented in Table 3. In both Model 1 (the DM model) and Model 2 (the DM with CVD model), the prediction results indicated that the DM population would increase rapidly, and the prediction results of the two models remained consistent. CVD variables were introduced in Model 2. The results indicated that the growth of DM patients without CVD would be slow, but the growth of DM patients with CVD would be rapid. These data indicate that by 2050, the number of patients with DM in the Chinese population will increase rapidly, and the growth rate of patients with DM and CVD will accelerate. CVD would become the main complication of diabetes.

**TABLE 3:** Simulated values of population with diabetes in different scenario from 2020 to 2050 (System dynamic model simulates the growth trend of diabetes mellitus, China. 2020–2050).

Predicted population (Million)	Model 1	Model 2	DM none CVD	DM with CVD
	DM	DM		
Baseline data in 2007	92.40	92.40	61.08	31.32
No intervention				
2020	141.65 (Ref)	140.94 (Ref)	75.36 (Ref)	65.58 (Ref)
2025	153.90 (Ref)	153.14 (Ref)	75.90 (Ref)	77.25 (Ref)
2035	174.89 (Ref)	173.96 (Ref)	76.24 (Ref)	97.71 (Ref)
2050	202.84 (Ref)	201.09 (Ref)	78.21 (Ref)	122.88 (Ref)
2025 vs. 2020%	8.65	8.66	0.72	17.80
2035 vs. 2020%	23.47	23.43	1.17	48.99
2050 vs. 2020%	43.20	42.68	3.78	87.37
Lifestyle therapy				
2020 (vs. Ref %)	119.65 (−15.53)	117.30 (−16.77)	73.20 (−2.87)	44.10 (−32.75)
2025 (vs. Ref %)	127.68 (−17.04)	124.76 (−18.53)	75.91 (+0.02)	48.86 (−36.76)
2035 (vs. Ref %)	142.46 (−18.55)	138.47 (−20.40)	80.55 (+5.65)	57.92 (−40.72)
2050 (vs. Ref %)	163.57 (−19.36)	157.78 (−21.54)	87.28 (+11.60)	70.50 (−42.63)
2025 vs. 2020%	6.71	6.36	3.71	10.79
2035 vs. 2020%	19.06	18.05	10.04	31.34
2050 vs. 2020%	36.71	34.51	19.23	59.86
Metformin therapy				
2020 (vs. Ref %)	125.32 (−11.53)	124.97 (−11.33)	70.07 (−7.02)	54.90 (−16.29)
2025 (vs. Ref %)	134.45 (−12.64)	134.08 (−12.45)	71.08 (−6.35)	62.99 (−18.46)
2035 (vs. Ref %)	150.79 (−13.78)	150.24 (−13.64)	72.68 (−4.67)	77.56 (−20.62)
2050 (vs. Ref %)	173.50 (−14.46)	172.16 (−14.39)	75.92 (−2.93)	96.24 (−21.68)
2025 vs. 2020%	7.29	7.29	1.44	14.74
2035 vs. 2020%	20.32	20.22	3.72	41.28
2050 vs. 2020%	38.45	37.76	8.35	75.30
Acarbose therapy				
2020 (vs. Ref %)	125.81 (−11.18)	124.71 (−11.52)	73.45 (−2.53)	51.26 (−21.84)
2025 (vs. Ref %)	134.20 (−12.80)	133.07 (−13.11)	74.84 (−1.40)	58.23 (−24.62)
2035 (vs. Ref %)	148.86 (−14.88)	147.73 (−15.08)	76.88 (+0.84)	70.85 (−27.49)
2050 (vs. Ref %)	169.16 (−16.60)	167.79 (−16.56)	80.63 (+3.09)	87.16 (−29.07)
2025 vs. 2020%	6.67	6.70	1.89	13.60
2035 vs. 2020%	18.32	18.46	4.67	38.22
2050 vs. 2020%	34.46	34.54	9.78	70.04
Voglibose therapy				
2020 (vs. Ref %)	112.67 (−20.46)	116.93 (−17.04)	50.13 (−33.48)	66.79 (+1.85)
2025 (vs. Ref %)	116.88 (−24.05)	122.25 (−20.17)	46.24 (−39.08)	76.01 (−1.61)
2035 (vs. Ref %)	124.27 (−28.94)	131.59 (−24.36)	41.49 (−45.58)	90.09 (−7.80)
2050 (vs. Ref %)	135.51 (-33.19)	145.16 (−27.81)	39.77 (−49.15)	105.39 (−14.23)
2025 vs. 2020%	3.74	4.55	−7.76	13.80
2035 vs. 2020%	10.30	12.54	−17.24	34.89
2050 vs. 2020%	20.27	24.14	−20.67	57.79

DM, diabetes mellitus; CVD, cardiovascular disease. Models 1 and 2 refer to diabetes and diabetes combined with CVD, respectively.

When four intervention scenarios, namely, were lifestyle intervention and the use of metformin, acarbose, and voglibose, were introduced into both Model 1 and Model 2, the predicted number of DM population decreased (Table 3). All four interventions can reduce the number of DM population with CVD.

### Projections of DM Intervention Cost

The simulation results for the impact of the intervention on the total cost of diabetes are presented in Table 4. The results from Model 1 indicated that by 2050, compared with 2020, the annual cost for the entire DM population in China would increase by 43.19%, and the total cost would reach 182.55 billion. In Model 1, lifestyle intervention and the use of metformin and acarbose would increase the annual cost for the entire DM population. However, the use of voglibose would decrease the annual cost. These results indicated that the use of voglibose could reduce the overall treatment burden of the DM population. In Model 2, when CVD was introduced, the overall cost of the DM population increased significantly, which was mainly due to the increased cost of CVD prevention and treatment. Lifestyle intervention and the use of metformin and acarbose would increase the annual cost for the DM population without CVD but could reduce the overall cost of the DM population. Interestingly, all four interventions could reduce the cost of the DM population with CVD.

**TABLE 4:** Simulated values of cost in different scenario from 2020 to 2050 (System dynamic model simulates the growth trend of diabetes mellitus, China. 2020–2050).

Predicted Cost (US$, Billion)	Model 1	Model 2	DM none CVD	DM with CVD
	DM	DM		
No intervention				
2020	127.49 (Ref)	263.11 (Ref)	67.83 (Ref)	195.29 (Ref)
2025	138.51 (Ref)	298.35 (Ref)	68.31 (Ref)	230.05 (Ref)
2035	157.40 (Ref)	359.61 (Ref)	68.62 (Ref)	290.99 (Ref)
2050	182.55 (Ref)	436.32 (Ref)	70.39 (Ref)	365.93 (Ref)
2025 vs. 2020%	8.64	13.39	0.71	17.80
2035 vs. 2020%	23.46	36.68	1.16	49.01
2050 vs. 2020%	43.19	65.83	3.77	87.38
Lifestyle therapy				
2020 (vs. Ref %)	152.07 (+19.28)	240.74 (−8.50)	93.03 (+37.15)	147.71 (−24.36)
2025 (vs. Ref %)	162.28 (+17.16)	260.10 (−12.82)	96.48 (+41.24)	163.62 (−28.88)
2035 (vs. Ref %)	181.06 (+15.03)	296.36 (-17.59)	102.38 (+49.20)	193.97 (−33.34)
2050 (vs. Ref %)	207.90 (+13.89)	347.04 (−20.46)	110.93 (+57.59)	236.10 (−35.48)
2025 vs. 2020%	6.71	8.04	3.71	10.77
2035 vs. 2020%	19.06	23.10	10.05	31.32
2050 vs. 2020%	36.71	44.16	19.24	59.84
Metformin therapy				
2020 (vs. Ref %)	133.21 (+4.49)	246.93 (−6.15)	74.49 (+9.82)	172.44 (−11.70)
2025 (vs. Ref %)	142.92 (+3.18)	273.42 (−8.36)	75.56 (+10.61)	197.86 (−13.99)
2035 (vs. Ref %)	160.29 (+1.84)	320.87 (−10.77)	77.26 (+12.59)	243.61 (−16.28)
2050 (vs. Ref %)	184.43 (+1.03)	383.01 (−12.22)	80.70 (+14.65)	302.30 (−17.39)
2025 vs. 2020%	7.29	10.73	1.44	14.74
2035 vs. 2020%	20.33	29.94	3.72	41.27
2050 vs. 2020%	38.45	55.11	8.34	75.31
Acarbose therapy				
2020 (vs. Ref %)	136.51 (+7.08)	241.82 (−8.09)	79.69 (+17.48)	162.12 (−16.98)
2025 (vs. Ref %)	145.61 (+5.13)	265.37 (−11.05)	81.20 (+18.87)	184.17 (−19.94)
2035 (vs. Ref %)	161.52 (+2.62)	307.51 (−14.49)	83.42 (+21.57)	224.09 (−22.99)
2050 (vs. Ref %)	183.54 (+0.54)	363.18 (−16.76)	87.48 (+24.28)	275.70 (−24.66)
2025 vs. 2020%	6.67	9.74	1.89	13.60
2035 vs. 2020%	18.32	27.16	4.68	38.22
2050 vs. 2020%	34.45	50.19	9.78	70.06
Voglibose therapy				
2020 (vs. Ref %)	128.67 (+0.93)	272.33 (+3.50)	57.25 (−15.60)	215.08 (+10.13)
2025 (vs. Ref %)	133.47 (−3.64)	297.55 (−0.27)	52.80 (−22.71)	244.75 (+6.39)
2035 (vs. Ref %)	141.92 (−9.83)	337.49 (−6.15)	47.38 (−30.95)	290.10 (−0.31)
2050 (vs. Ref %)	154.76 (−15.22)	384.77 (−11.81)	45.41 (−35.49)	339.35 (−7.26)
2025 vs. 2020%	3.73	9.26	−7.77	13.79
2035 vs. 2020%	10.30	23.93	−17.24	34.88
2050 vs. 2020%	20.28	41.29	−20.68	57.78

DM, diabetes mellitus; CVD, cardiovascular disease. Models 1 and 2 refer to diabetes and diabetes combined with CVD, respectively.

## Discussion

“Joint construction, sharing and health for all” is the strategic theme of building a healthy China. China is a vast country, and the health strategy is formulated by the country. In 2016, China issued the outline of the “healthy China 2030” plan and listed the full coverage of management interventions for patients with diabetes. Due to the lack of prediction models for future development trends in the DM population in China, it remains still unclear how to formulate future prevention and treatment strategies for DM in China. Establishing SD model is a way to solve this problem. Jones AP et al. [23] developed a SD model to explain the growth of diabetes since 1980 through 2050 in the United States. Three policy intervention scenarios, which were enhanced clinical management of diabetes, increased management of prediabetes, and reduced obesity prevalence, were evaluated [23]. Edwards RA et al. [39] reported a comprehensive causal simulator by using SD methodology to identify leverage points among diabetes interventions in the US. Sluijs T et al. [7] used the SD modeling methodology to develop a simulation model about the patient journey of type 2 DM and to assess the impact of lifestyle intervention programs on total cost for society in the Netherlands. Freebairn L et al. [40] developed dynamic simulation models to explore the impact of maternal weight status interventions on incidence of hyperglycemia in pregnancy. Ansah JP et al. [22] developed a SD model to evaluate the impact of hypertension, diabetes and smoking cessation management on CVD outcomes. Sugiyama T et al. [41] developed a SD model to predict the number of people with diabetes and initiation of dialysis due to diabetic nephropathy in Japan. The hypothetical interventions in their study were glycemic control, blood pressure control, and protein and salt reduction [41]. Guariguata L et al. [42] developed a diabetes SD model for guiding policy on diabetes in the Caribbean. Compared with these studies, we did not distinguish the type of diabetes, and the study population in our study included both type 1 diabetes and type 2 diabetes. This is mainly because the four diabetes epidemiological data in China included in our study did not distinguish the type of diabetes. In addition, CVD complications were added to our Model 2, which was not involved in previous study. In this study, we developed two DM prevention and control models to predict the number of people with DM and the economic burden of controlling DM. The results of both models suggest that the DM population in China will increase significantly by 2050. Diabetes-related health expenditures in China are projected to reach 182.55 billion USD by 2050. When CVD treatment was considered, the expenditure was 1.5–2.5-fold higher. Therefore, establishing a prediction model or DM in the Chinese population is important for predicting health intervention needs and planning public health plans.

To date, only two studies have addressed the projection of patients with DM in China. The IDF Diabetes Atlas 10th edition estimates that in 2021, 140.9 million people will be living with diabetes in China, which will increase to 174.4 million by 2045 [5]. However, their study did not include large national survey data in China when predicting the DM population and did not predicted for the year 2050. A recent Chinese study used the autoregressive integrated moving average (ARIMA) model to estimate the DM population and its economic burden from 2020 to 2025 [43]. This model predicted that by 2025, the DM population in China would be approximately 100 million and the financial burden of diabetes would be nearly 170 billion. However, the large national survey indicated that the total number of patients with diabetes in China was estimated to be 113.9 million in 2010 and 129.8 million in 2017 [4, 30]. The above ARIMA model only assessed a relatively short time horizon prediction, and the projection of the DM population was far from the published prevalence studies. Our study not only included the main national survey data but also extended the simulation time horizon from 2020 to 2050. In addition, we used the SD method to construct two sub-models of the diabetes journey, diabetes journey and diabetes with CVD journey. Notably, this study improved our understanding of the characteristic dynamics of the simulated DM population in China.

In this study, we predicted from Model 1 that by 2025, the DM population in China would be 153.90 million, which is higher than the 100 million estimated in a previous study [43]. We also predicted that by 2035, 174.89 million people in China would be diabetic, 10 years ahead of the previous study, which predicted that there would be 174.4 million diabetic people in 2045 [5]. The differences between our estimates and those of previous studies could be attributed to several factors. First, our initial population with diabetes, which was derived from national survey data, was higher than that predicted by previous studies. However, the initial predicted population with diabetes in 2007 was 92.4 million according to a national survey from 14 provinces and municipalities, which was used to simulate our study [8]. Second, we used four national surveys conducted between 2007 and 2017 to validate the results, and the model was considered valid and reasonable [4, 8, 30, 31]. In addition, we adopted SD to simulate our model, which can simulate nonlinear relationships between components of the systems, resulting in behaviors such as feedback loops and long delays between cause and effect, and the behaviors can change dynamically.

The State Council of China endorsed an important document as a response to disease epidemics, the Medium-to Long-term Plan for the Prevention and Treatment of Chronic Diseases (2017–2025), which aimed to decrease the mortality rate of CVD by 15% by 2025 compared with 2015 in China [44]. The CAPTRU study estimated that the prevalence of CVD in patients with diabetes in China was 33.9% [45]. CVD is the leading cause of diabetes-related morbidity and mortality. The glycated hemoglobin level is reduced by 0.9%, and major cardiovascular events can be reduced by 33% [46]. In Model 2, the predicted total DM population was consistent with that of Model 1. However, we estimated that there would a rapid growth in the number of diabetic patients with CVD from 2020 to 2050, and the predicted DM population would be 122.88 million in 2050. Therefore, the prevention and treatment of diabetes play an important role in reducing the incidence of cardiovascular complications.

Lifestyle therapy and the use of metformin and acarbose are the main strategies for the prevention of prediabetes [29]. In a cross-sectional study of type 2 diabetes, more than 50% patients had at least one chronic diabetes complication, with a prevalence of cardiovascular and cerebrovascular complications of 30.1% and 6.8%, respectively [47]. As α-glucosidase inhibitors confer a cardiovascular risk benefit, we introduced another α-glucosidase inhibitor, voglibose, into this model and predicted its effect on controlling DM [16]. After using the four scenarios for model simulation, the results indicated that by 2050, the overall DM population and DM patients with CVD would be significantly reduced. The prediction results showed that lifestyle therapy and the use of voglibose were associated with a remarkable decrease in the total number of patients with DM. However, only the use of metformin and voglibose can reduce the DM population without CVD in Model 2. In the two scenarios of lifestyle therapy and the use of acarbose, the prediction of DM population without CVD will still increase. This result can be attributed to the characteristics of the study population in the included studies. The role of lifestyle therapy and the use of acarbose in preventing diabetes with CVD is clear [1]. These studies about the lifestyle therapy and the use of acarbose included in our models were also concentrated in the population with DM combined with CVD. Therefore, in model 2, these two scenarios have little impact on the DM population without CVD.

The rapid growth of the DM population is accompanied by an increase in the economic burden. In our study, when the cost of CVD treatment was not considered, the predicted annual economic burden for the entire DM population in China was 182.55 billion by 2050. As many patients with DM have complications, the results from Model 1 may underestimate the real economic burden of the DM population. When considering CVD, the annual economic burden for the entire DM population was predicted to be 436.32 billion by 2050. The expenditure simulated by Model 2 was 1.5–2.5-fold higher than that of Model 1 in each scenario. These results suggest that, in the process of diabetes intervention, interventions should be fully considered for patients with CVD.

Based on the results of this study, we make the following public health and policy recommendations for policymakers: First, population with DM in China is predicted to grow rapidly, and active measures should be taken to intervene in the progress of this population. Lifestyle therapy and drug intervention maybe effectively slow the growth of the population with DM in China. In the future, China should strengthen the guidance and science popularization of the healthy lifestyle of people with DM and people at risk of DM, and actively promote lifestyle therapy and drug intervention. Second, China will face severe challenges of DM combined with CVD in the future. Our results predict that lifestyle therapy and drug intervention can effectively slow the growth and economic burden of people with DM combined with CVD. At present, acarbose is the only α-glycosidase inhibitor among the national essential drugs. It is recommended that voglibose be included in national essential drugs. Third, the government should strengthen the scientific prediction of the growth of the DM population in China and pay more attention to DM combined diseases, establish a more comprehensive SD model around DM and its combined diseases, and multiple treatment and intervention measures of DM, study and judge DM prevention and treatment situation and future development trend in China, and issue relevant policies in a timely manner.

### Strengths and Limitations

The main strength of this study is that we combined recent national survey data to project the total population with diabetes using SD models.

However, our model has several limitations. First, although a national survey and large prevention studies were used, some data estimates and assumptions were made due to limited data. Second, no distinction in age groups was made, because specific data for all other variables per age group were scarce. Third, China is a vast country, but we did not study rural/urban differences. Furthermore, we only included CVD among the combined diseases of DM and did not include other diseases such as obesity. Finally, only four interventions were included in our models, and other strategies, such as the use of new hypoglycemic drugs, including sodium-dependent glucose transporter 2 inhibitors, were not included in this study.

### Conclusion

Our study shows that the SD model can be used to predict the number of DM population in China. The projection of the DM population rapidly increases from 2020 to 2050, and this situation is serious. Lifestyle therapy and the use of hypoglycemic drugs, such as metformin and acarbose, could delay the growth of the DM population and reduce the economic burden. Our models predicted that voglibose would also have a postitve effect on controlling the growth of the DM population.

## Data Availability

The data supporting the findings of this study are available upon reasonable request from the corresponding authors.
