# Supplementary material for: System Dynamic Model Simulates the Growth Trend of Diabetes Mellitus in Chinese Population: Implications for Future Urban Public Health Governance
Source: Int J Public Health. 2022 Nov 11;67:1605064. doi: 10.3389/ijph.2022.1605064 (PMC9691669; doi:10.3389/ijph.2022.1605064)
Supplement: Supplementary file 1 [file DataSheet1.docx]

**Table S1: The characteristics data and variables of diabetes patient journey (System dynamic model simulates the growth trend of diabetes mellitus, China. 2020-2050)**

| Diabetes patient Journey | | | |
| --- | --- | --- | --- |
| Model setting data variables | | | |
| Start time | 2007 |  |  |
| Stop time | 2050 |  |  |
| Time step (dT) | 1 |  |  |
| Fractional DT | TRUE |  |  |
| Save interval | 1 |  |  |
| Time units | years |  |  |
| Integration method | Euler |  |  |
| Data and equation in the model | | | |
| Initial data, progression rate or incidence rate in the model | | | |
| Variables | Data | Units or interpretations | References |
| INIT diabetes population | 9240 | Ten thousand | ^1^ |
| INIT prediabetes population | 14820 | Ten thousand | ^1^ |
| INIT normoglycemic population | 71198 | Ten thousand | ^1^ |
| INIT no medicine necessary | 0 | Ten thousand | ^2^ |
| Chinese population in 2007 | 132129 | Ten thousand | China population census |
| Rate of diabetes diagnoses | 0.367 | Also refer to diagnoses of prediabetes | ^1^ |
| Normoglycemic population to pre-diabetes (non-obesity) | 0.01 | 2 | ^2^ |
| Normoglycemic population to pre-diabetes (obesity) | 0.02905 | Risk ratio (RR) 5.81  Obesity vs. non-obesity | ^3^ |
| Incidence of obesity | 0.333 |  | ^4^ |
| No medicine necessary to diabetes | 0.083 | 1 | ^5^ |
| Undiagnosed prediabetes to diabetes | 0.083 | 1 | ^2,5^ |
| Diagnosed prediabetes to diabetes | 0.113 | 1 | ^2,5^ |
| Diabetes recovered to no medicine necessary | 0.001 | 3 | ^2^ |
| Undiagnosed prediabetes to normoglycemic population | 0.322 | 6 | ^6^ |
| Diagnosed prediabetes to normoglycemic population | 0.322 | 6 | ^6^ |
| Death rate | 0.0170 |  | ^5^ |
| Death rate of prediabetes | 0.0073 | RR 1.13 prediabetes vs. normal | ^7^ |
| Death rate of non-diabetes | 0.00646 |  | ^8^ |
| Birth rate in 2007 | 0.0121 |  | China population census |
| Diagnosed rate pre-or diabetes | 0.4124 |  | ^1^ |
| Undiagnosed rate pre-or diabetes | 0.5876 |  | ^1^ |
| Equation |  |  |  |
| Stocks | | | |
| Normoglycemic population | INTEG (Birth population per year+No medicine necessary to normoglycemic population+Undiagnosed prediabetes to normoglycemic population+Diagnosed prediabetes to normoglycemic population-Normoglycemic population to undiagnosed prediabetes-Normoglycemic population to diagnosed prediabetes-Normoglycemic death population per year) Initial Value 71198 | | |
| No medicine necessary population | INTEG (Diabetes to no medicine necessary pupulation-No medicine necessary to Diabetes-No medicine necessary to normoglycemic population) Initial Value 0 | | |
| Undiagnosed prediabetes population | INTEG (Normoglycemic population to undiagnosed prediabetes-Undiagnosed prediabetes to Diabetes-Undiagnosed prediabetes to normoglycemic population-Undiagnosed prediabetes death population) Initial Value 8708 | | |
| Diagnosed prediabetes population | INTEG (Normoglycemic population to diagnosed prediabetes-Diagnosed prediabetes to Diabetes-Diagnosed prediabetes to normoglycemic population-Diagnosed prediabetes death population) Initial Value 6112 | | |
| Diabetes population | INTEG (No medicine necessary to diabetes+ Undiagnosed prediabetes to diabetes+ Diagnosed prediabetes to diabetes-diabetes to no medicine necessary population-diabetes death population) Initial Value 9240 | | |
| Flows | | | |
| Normoglycemic death population per year | Normoglycemic population*Death rate 4 | | |
| Birth population per year | Chinese population*Birth rate | | |
| No medicine necessary to normoglycemic population | No medicine necessary population*Recovery rate 5/Average time to recovery 5 | | |
| Undiagnosed prediabetes to normoglycemic population | Undiagnosed prediabetes population*Recovery rate/Average time to recovery | | |
| Normoglycemic population to undiagnosed prediabetes | Undiagnosed rate* Normoglycemic population*Incidence rate/Average time to occurrence | | |
| Normoglycemic population to diagnosed prediabetes | Diagnosed rate* Normoglycemic population*Incidence rate 1/Average time to occurrence 1 | | |
| Diagnosed prediabetes to normoglycemic population | Diagnosed prediabetes population*Recovery rate 1/Average time to recovery 1 | | |
| Diabetes to no medicine necessary pupulation | Diabetes population*Recovery rate 4/Average time to recovery 4 | | |
| No medicine necessary to diabetes | No medicine necessary population*Incidence rate 4/Average time to occurrence 4 | | |
| Undiagnosed prediabetes to diabetes | Undiagnosed rate*Undiagnosed prediabetes population*Incidence rate 2/Average time to occurrence 2 | | |
| Diagnosed prediabetes to diabetes | Diagnosed rate*Diagnosed prediabetes population*Incidence rate 3/Average time to occurrence 3 | | |

**Table S2: The characteristics data and variables of diabetes combined cardiovascular diseases patient journey (System dynamic model simulates the growth trend of diabetes mellitus, China. 2020-2050)**

| Diabetes combined CVD patient journey | | | |
| --- | --- | --- | --- |
| Model setting data variables | | | |
| Start time | 2007 |  |  |
| Stop time | 2050 |  |  |
| Time step (dT) | 1 |  |  |
| Fractional DT | TRUE |  |  |
| Save interval | 1 |  |  |
| Time units | years |  |  |
| Integration method | Euler |  |  |
| Data and equation in the model | | | |
| Initial data, progression rate or incidence rate in the model | | | |
| Variables | Data | Units or interpretations | References |
| INIT diabetes population | 9240 | Ten thousand | ^1^ |
| INIT diabetes population without CVD | 9240*96.17%=  8886 | Ten thousand | ^1,9^ |
| INIT diabetes population with CVD | 9240*3.83%=  354 | Ten thousand | ^1,9^ |
| INIT prediabetes population | 14820 | Ten thousand | ^1^ |
| INIT normoglycemic population | 71198 | Ten thousand | ^1^ |
| INIT no medicine necessary | 0 | Ten thousand | ^2^ |
| Chinese population in 2007 | 132129 | Ten thousand | China population census |
| Rate of diabetes diagnoses | 0.367 | Also refer to diagnoses of prediabetes | ^1^ |
| Normoglycemic population to pre-diabetes (non-obesity) | 0.01 | 2 | ^2^ |
| Normoglycemic population to pre-diabetes (obesity) | 0.02905 | Relative risks (RR) 5.81  Obesity vs. non-obesity | ^3^ |
| Incidence of obesity | 0.333 |  | ^4^ |
| No medicine necessary to diabetes without CVD | 0.083 | 1 | ^2,5^ |
| Undiagnosed prediabetes to diabetes without CVD | 0.083 | 1 | ^2,5^ |
| Diabetes recovered to no medicine necessary | 0.001 | 3 | ^2^ |
| Undiagnosed prediabetes to normoglycemic population | 0.322 | 6 | ^6^ |
| Diagnosed prediabetes to normoglycemic population | 0.322 | 6 | ^6^ |
| Death rate of diabetes without CVD | 0.0170 |  | ^5^ |
| Death rate of prediabetes | 0.0073 | RR 1.13 prediabetes vs. normal | ^7^ |
| Death rate of non-diabetes | 0.00646 |  | ^8^ |
| Birth rate in 2007 | 0.0121 |  | China population census |
| Prediabetes to diabetes with CVD rate | 0.0259 |  | ^6^ |
| Prediabetes to diabetes without CVD rate | 0.0739 |  | ^5,6^ |
| Diabetes without CVD to CVD rate | 0.0391 |  | ^6^ |
| Death rate of diabetes with CVD | 0.0195 |  | ^10^ |
| Equation |  |  |  |
| Stocks | | | |
| Normoglycemic population | INTEG (Birth population per year+Diagnosed prediabetes to normoglycemic population+No medicine necessary to normoglycemic population+Undiagnosed prediabetes to normoglycemic population-Normoglycemic death population per year-Normoglycemic population to diagnosed prediabetes-Normoglycemic population to undiagnosed prediabetes) Initial Value 71198 | | |
| No medicine necessary population | INTEG (Diabetes to no medicine necessary pupulation-No medicine necessary to diabetes-No medicine necessary to normoglycemic population) Initial Value 0 | | |
| Undiagnosed prediabetes population | INTEG (Normoglycemic population to undiagnosed prediabetes-Undiagnosed prediabetes to diabetes-Undiagnosed prediabetes to normoglycemic population-Undiagnosed prediabetes death population) Initial Value 8708 | | |
| Diagnosed prediabetes population | INTEG (Normoglycemic population to diagnosed prediabetes-Diagnosed prediabetes to diabetes-Diagnosed prediabetes to normoglycemic population-Diagnosed prediabetes death population) Initial Value 6112 | | |
| Diabetes population without CVD | INTEG (Diagnosed prediabetes to diabetes+ No medicine necessary to diabetes+ Undiagnosed prediabetes to diabetes-Diabetes death population-Diabetes to no medicine necessary population-Without CVD to diabetes with CVD) Initial Value 8886 | | |
| Diabetes population with CVD | INTEG (Prediabetes to diabetes with CVD+ Without CVD to diabetes with CVD-Diabetes with CVD death population) Initial Value 354 | | |
| Flows | | | |
| Normoglycemic death population per year | Normoglycemic population*Death rate 4 | | |
| Birth population per year | Chinese population*Birth rate | | |
| No medicine necessary to normoglycemic population | No medicine necessary population*Recovery rate 5/Average time to recovery 5 | | |
| Undiagnosed prediabetes to normoglycemic population | Undiagnosed prediabetes population*Recovery rate/Average time to recovery | | |
| Normoglycemic population to undiagnosed prediabetes | Undiagnosed rate*Normoglycemic population*Incidence rate/Average time to occurrence | | |
| Normoglycemic population to diagnosed prediabetes | Diagnosed rate* Normoglycemic population*Incidence rate 1/Average time to occurrence 1 | | |
| Diagnosed prediabetes to normoglycemic population | Diagnosed prediabetes population*Recovery rate 1/Average time to recovery 1 | | |
| Diabetes to no medicine necessary population | Diabetes population*Recovery rate 4/Average time to recovery 4 | | |
| No medicine necessary to diabetes | No medicine necessary population*Incidence rate 4/Average time to occurrence 4 | | |
| Undiagnosed prediabetes to diabetes | Undiagnosed rate*Undiagnosed prediabetes population*Incidence rate 2/Average time to occurrence 2 | | |
| Diagnosed prediabetes to diabetes | Diagnosed rate*Diagnosed prediabetes population*Incidence rate 3/Average time to occurrence 3 | | |
| Prediabetes to diabetes with CVD | Diagnosed rate*Diagnosed prediabetes population*CVD incidence rate 2 | | |
| Normal to diabetes with CVD population | Normoglycemic population*CVD incidence rate 1 | | |
| Without CVD to diabetes with CVD | Diabetes population without CVD*CVD incidence rate 3 | | |

**Table S3: The characteristics data and variables of the diabetes cost journey (System dynamic model simulates the growth trend of diabetes mellitus, China. 2020-2050)**

| Diabetes cost Journey | | | |
| --- | --- | --- | --- |
| Model setting data variables | | | |
| Start time | 2007 |  |  |
| Stop time | 2050 |  |  |
| Time step (dT) | 1 |  |  |
| Fractional DT | TRUE |  |  |
| Save interval | 1 |  |  |
| Time units | years |  |  |
| Integration method | Euler |  |  |
| Data and equation in the model | | | |
| Initial data, progression rate or incidence rate in the model | | | |
| Variables | Data | Units or interpretations | References |
| INIT cost of screening | 3 | US dollar | ^11^ |
| INIT onset of diabetes | 897 | US dollar | ^12^ |
| INIT CVD treatment | 2078 | US dollar | ^12^ |
| INIT Lifestyle intervention | 371 | US dollar | ^11^ |
| INIT Metformin intervention | 163 | US dollar | ^13^ |
| INIT Acarbose intervention | 185 | US dollar | ^14^ |
| INIT Voglibose intervention | 242 | US dollar | ^15^ |
| Equation |  |  |  |
| Stocks | | | |
| Total fixed cost per diabetes patient | INTEG (Cost of screening+Onset of diabetes) Initial Value 900 | | |
| Total fixed cost per diabetes patient | INTEG (Cost of screening+Onset of diabetes+ cardiovascular diseases treatment) Initial Value 2978 | | |
| Total variable cost per diabetes patient | INTEG (No intervention: OR: Total costs life style intervention per diabetes patient: OR: Total costs of acarbose intervention per diabetes patient: OR: Total costs of metformin intervention per diabetes patient: OR: Total costs of voglibose intervention per diabetes patient )  Initial Value 0 | | |
| Total cost of diabetes | INTEG (Total fixed cost per diabetes patient*diabetes population+Total variable cost per diabetes patient*diabetes population) Initial Value 83.16 | | |
| Flows | | | |
| Cost of screening | 2-hour postprandial blood glucose+Oral glucose tolerance test | | |
| Onset of diabetes | Laboratory tests cost 1+Drug treatment of diabetes | | |
| Cardiovascular diseases treatment | Drug treatment of diabetes+Drug treatment of CVD+ Laboratory tests costs 2 | | |

**Figure S1: A schematic overview of the total costs of diabetes using dynamic simulations (System dynamic model simulates the growth trend of diabetes mellitus, China. 2020-2050)**


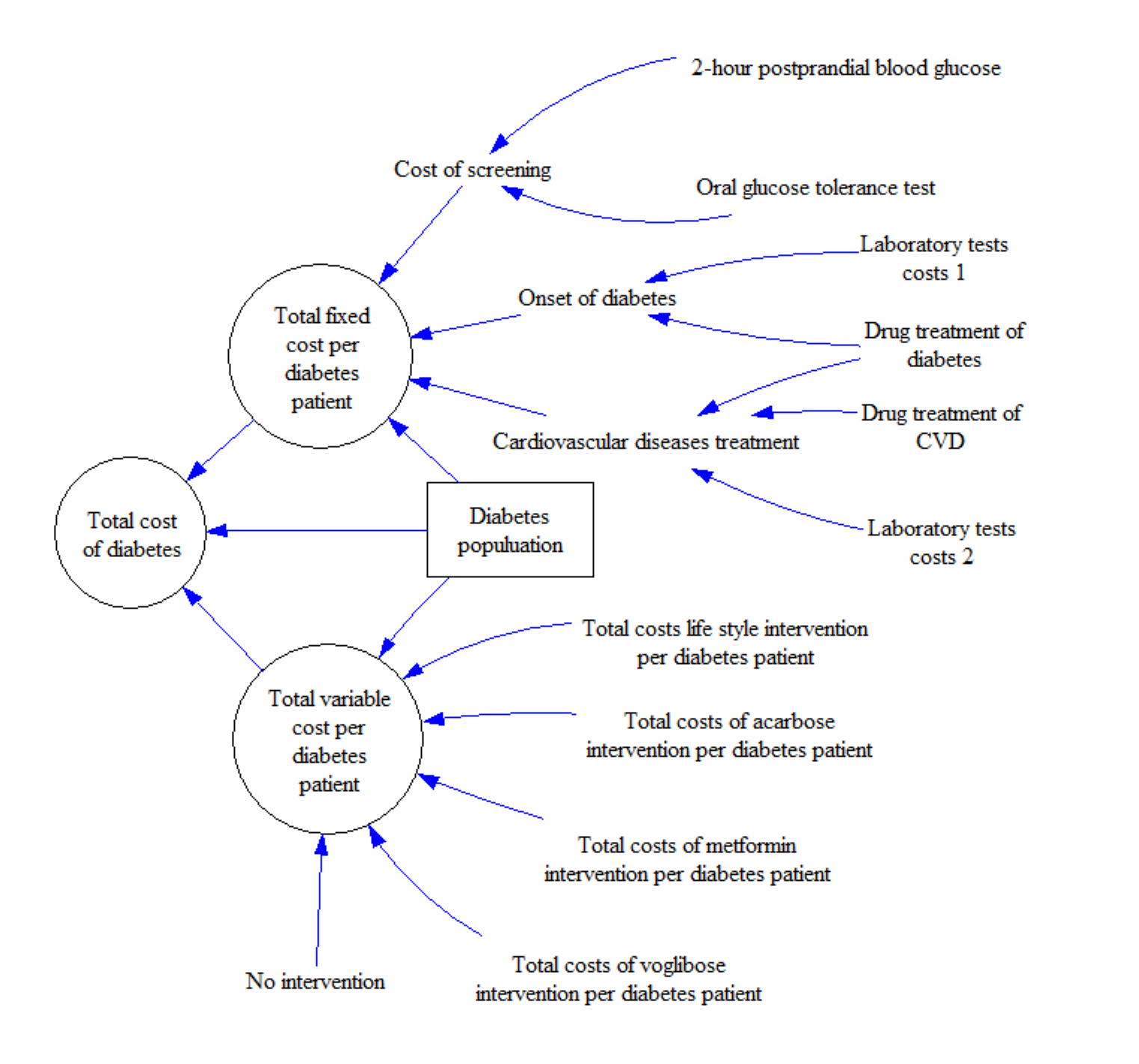


**References**

1. Yang W, Lu J, Weng J, et al. Prevalence of diabetes among men and women in China. *The New England journal of medicine.* 2010;362(12):1090-1101.

2. Sluijs T, Lokkers L, Özsezen S, Veldhuis GA, Wortelboer HM. An Innovative Approach for Decision-Making on Designing Lifestyle Programs to Reduce Type 2 Diabetes on Dutch Population Level Using Dynamic Simulations. *Frontiers in public health.* 2021;9:652694.

3. Schnurr TM, Jakupović H, Carrasquilla GD, et al. Obesity, unfavourable lifestyle and genetic risk of type 2 diabetes: a case-cohort study. *Diabetologia.* 2020;63(7):1324-1332.

4. Zhang L, Wang Z, Wang X, et al. Prevalence of overweight and obesity in China: Results from a cross-sectional study of 441 thousand adults, 2012-2015. *Obesity research & clinical practice.* 2020;14(2):119-126.

5. Li G, Zhang P, Wang J, et al. The long-term effect of lifestyle interventions to prevent diabetes in the China Da Qing Diabetes Prevention Study: a 20-year follow-up study. *Lancet (London, England).* 2008;371(9626):1783-1789.

6. Chen Y, Zhang P, Wang J, et al. Associations of progression to diabetes and regression to normal glucose tolerance with development of cardiovascular and microvascular disease among people with impaired glucose tolerance: a secondary analysis of the 30 year Da Qing Diabetes Prevention Outcome Study. *Diabetologia.* 2021;64(6):1279-1287.

7. Huang Y, Cai X, Mai W, Li M, Hu Y. Association between prediabetes and risk of cardiovascular disease and all cause mortality: systematic review and meta-analysis. *BMJ (Clinical research ed).* 2016;355:i5953.

8. Bragg F, Holmes MV, Iona A, et al. Association Between Diabetes and Cause-Specific Mortality in Rural and Urban Areas of China. *Jama.* 2017;317(3):280-289.

9. Yang Z, Xing X, Xiao J, et al. Prevalence of cardiovascular disease and risk factors in the Chinese population with impaired glucose regulation: the 2007-2008 China national diabetes and metabolic disorders study. *Experimental and clinical endocrinology & diabetes : official journal, German Society of Endocrinology [and] German Diabetes Association.* 2013;121(6):372-374.

10. Neal B, Perkovic V, Mahaffey KW, et al. Canagliflozin and Cardiovascular and Renal Events in Type 2 Diabetes. *The New England journal of medicine.* 2017;377(7):644-657.

11. Liu X, Li C, Gong H, et al. An economic evaluation for prevention of diabetes mellitus in a developing country: a modelling study. *BMC public health.* 2013;13:729.

12. Wang W, McGreevey WP, Fu C, et al. Type 2 diabetes mellitus in China: a preventable economic burden. *The American journal of managed care.* 2009;15(9):593-601.

13. Knowler WC, Barrett-Connor E, Fowler SE, et al. Reduction in the incidence of type 2 diabetes with lifestyle intervention or metformin. *The New England journal of medicine.* 2002;346(6):393-403.

14. Chiasson JL, Josse RG, Gomis R, Hanefeld M, Karasik A, Laakso M. Acarbose for prevention of type 2 diabetes mellitus: the STOP-NIDDM randomised trial. *Lancet (London, England).* 2002;359(9323):2072-2077.

15. Kawamori R, Tajima N, Iwamoto Y, Kashiwagi A, Shimamoto K, Kaku K. Voglibose for prevention of type 2 diabetes mellitus: a randomised, double-blind trial in Japanese individuals with impaired glucose tolerance. *Lancet (London, England).* 2009;373(9675):1607-1614.
